# Chemical Pathology

**Published:** 1907-11

**Authors:** 


**Chemical Pathology.** Being a Discussion of General Pathology from the Standpoint of the Chemical Processes Involved. By **H. Gideon Wells,** Ph.D., M.D., Assistant Professor of Pathology in the University of Chicago and in Rush Medical College, Chicago. Octavo, 549 pages. Philadelphia and London: W. B. Saunders Company, 1907. (Cloth, $3.25 net.)

This is a book of 550 pages, of good type, well printed, and written in style free from the pedantic language that mars the pages of so many medical and chemical works of the present day. It is a clear scholarly arrangement of the facts and theories of modern pathology viewed from the chemical standpoint. No authentic or useful study has been omitted and the bibliography contains practically every necessary reference. Excessive bibliography frequently sacrifices much space that in topics so valuable and interesting should be devoted to the work in hand. This has been avoided by the painstaking author, while, at the same time his references are sufficient.

The book is a practical summary of modern knowledge of the subjects treated and it is so full of new and useful information that it is read with interest from cover to cover. It is a practical condensation of the various studies in this new field of medical research. It will be of great value to the student as it contains what he requires to know, in concise, clear language; to the practitioner it offers a guide to the sources of knowledge of these subjects and information of the latest of the various chemical processes that have so much to do with modern medical practice.

It is with rare skill that the many scattered fragments of knowledge have been brought together and correlated, so as to present in a field practically unexplored, a book that will prove a valuable textbook as well as a useful reference work for the library shelf.

